# The surgical outcomes and perioperative complications of bowel resection as part of debulking surgery of advanced ovarian cancer patients

**DOI:** 10.1186/s12893-022-01531-0

**Published:** 2022-03-04

**Authors:** Shuang Ye, Yiyong Wang, Lei Chen, Xiaohua Wu, Huijuan Yang, Libing Xiang

**Affiliations:** 1 Department of Gynecologic Oncology, Fudan University Shanghai Cancer Center, Shanghai, China; 2 Department of Oncology, Shanghai Medical College, Fudan University, Shanghai, China; 3 Department of Obstetrics and Gynecology, Baoshan Luodian Hospital, Shanghai, China; 4 Department of Radiology, Minhang Branch of Fudan University Shanghai Cancer Center, Shanghai, China; 5 Division of Gynecology Oncology, Department of Obstetrics and Gynecology, Zhongshan Hospital, Fudan University, Shanghai, China

**Keywords:** Bowel resection, Complications, Ovarian carcinoma, Surgical outcomes

## Abstract

**Background:**

To review the utilization of bowel resection in ovarian cancer surgery in our institution.

**Methods:**

All ovarian cancer patients who received bowel resection between 2006/01 and 2018/12 were identified. Postoperative morbidities were assessed according to the Clavien–Dindo classification (CDC).

**Results:**

There were 182 patients in the anastomosis group and 100 patients in the ostomy group, yielding a total of 282 patients. The median age was 57 years, and most patients had high-grade serous histology (88.7%). Forty-nine (17.3%) patients received neoadjuvant chemotherapy. During the operation, 78.7% of patients had ascites, and the median volume was 800 mL. Extensive bowel resection (at least two-segment) and upper abdominal operation were performed in 29 (10.2%) and 69 (24.4%) patients, respectively. The rectosigmoid colon was the most commonly resected (83.8%) followed by right hemicolectomy (5.9%) and small bowel resection (2.8%). No macroscopic residual disease was observed in 42.9% of the patients, whereas 87.9% had residual disease ≤ 1 cm. Among the entire cohort, 23.0% (65/282) experienced different complications. Severe complications (CDC 3–5) accounted for 9.2% of complications and were mostly categorized as pleural effusion requiring drainage (3.5%) followed by wound dehiscence requiring delayed repair in the operating room (1.8%). Nine patients experienced anastomotic leakage (AL): one in the ostomy group with extensive bowel resection and eight in the anastomosis group. The overall AL rate was 4.2% (9/212) per anastomosis.

**Conclusions:**

The execution of bowel resection as part of debulking surgery in patients with newly diagnosed ovarian cancer resulted in a severe morbidity rate of 9.2%.

## Background

Ovarian carcinoma is the most lethal gynaecologic malignancy [1]. Most patients present with advanced stage tumours, and optimal cytoreduction is well accepted to be the cornerstone of effective treatment in newly diagnosed patients [2, 3]. In the recurrent setting, surgery is also a valid option in patients fulfilling some criteria, such as no residual disease after primary surgery, good performance status, isolated recurrence, and platinum-sensitive ovarian recurrence [4]. In addition, even in platinum-resistant recurrence, experimental data demonstrated the role of surgery [5]. Debulking surgery for advanced ovarian cancer patients is complicated, requiring the removal of several organs and extensive amounts of peritoneum [6]. According to a recent publication from Japan, gynaecologic oncologists rarely perform bowel resection and upper abdominal operation [6], and the situation is similar in China. Only a few gynaecologic oncologists are willing to perform extensive radical surgery possibly due to either a lack of the relevant surgical skills or the intense patient–physician relationship [7].

As one of the leading cancer centres with high-volume cases, the gynaecologic oncologists in our department have adopted the concept of radical surgery, including upper abdominal surgery [7, 8] and modified posterior pelvic exenteration [9, 10]. In 2004, we first reported low colorectal staple anastomosis after rectosigmoid resection in primary surgery in eight ovarian cancer patients in a Chinese journal [9]. Then, a series of 50 cases between January 2006 and December 2010 was updated in 2018 [10]. The two publications were both in Chinese and exclusively focused on patients undergoing rectosigmoid resection and anastomosis. Both large and small bowels are involved in ovarian cancer patients with bulky tumours.

Numerous studies have focused on bowel surgery in ovarian cancer patients in Western countries [11–19]. In a study of 83 ovarian cancer patients who received bowel resections, important correlations were found between positive mesenteric, aortic and pelvic lymph nodes [18]. Therefore, radical bowel resection during debulking surgery for ovarian cancer patients with bowel involvement is recommended [18]. The authors also presented that pelvic posterior exenteration with retrograde radical hysterectomy has the benefit of good preservation of ladder and colorectal functions [14].

We conducted the current study to comprehensively review the utilization of bowel resection as part of debulking surgery in ovarian cancer patients. The specific details of bowel surgery and surgical-related outcomes were evaluated. A standardized scoring system was applied to assess perioperative complications.

## Methods

The study was approved by the Institutional Review Board, and the requirement for written informed consent was waived considering its retrospective design. We searched the electronic medical record database and included all patients with advanced ovarian cancer who underwent bowel surgery in primary or interval cytoreduction between January 2006 and December 2018 in our department.

Patient-, disease- and surgery-related information was abstracted from the medical records. The data collection included age at diagnosis, body mass index (BMI, calculated as weight (kg)/[height (m)]^2^), histology, and administration of neoadjuvant chemotherapy. Preoperative laboratory values, including haemoglobin, albumin, and cancer antigen 125 (CA-125), were also recorded. The following surgery-related variables were assessed: the presence and volume of ascites, upper abdominal surgery, type of bowel resection, estimated blood loss (EBL), intraoperative transfusion, extent of cytoreduction, postoperative complications, postoperative hospital stay and time interval from surgery to chemotherapy. A protective stoma was not routine and was performed at the surgeon’s discretion. The upper abdominal surgical procedures refer to the surgical cytoreduction of tumours in the upper abdominal region, which includes diaphragm peritonectomy/resection, splenectomy, and distal pancreatectomy [20, 21].

Patients who underwent a permanent ostomy formation were classified as the ostomy group even though some patients might have received multiple bowel resections and simultaneously underwent anastomosis and ostomy. The bowel resections were dichotomized into one-segment and extensive bowel resections (at least two-segment) [22]. The postoperative complications were graded according to the Clavien–Dindo classification (CDC) [23] and further categorized into the mild (CDC 0–2) and severe (CDC 3–5) subgroups [24]. All complications and CDC scores were recorded in patients who experienced more than one complication. We specifically focused on anastomotic leakage (AL) after bowel resection and anastomosis, which was defined as follows: (1) feculent fluid from the drainage tube, wound or vagina; (2) extravasation/leakage from the anastomotic site verified by imaging and/or intraoperative findings [16]. The AL rate was calculated both per patient and per anastomosis, considering that one patient might have at least two anastomoses after extensive bowel resection. Concerning the extent of debulking, R0 resection was defined as no visible gross tumour after cytoreduction, whereas R1 resection referred to residual disease ≤ 1 cm.

Statistical Package for Social Science (SPSS) (Version 17.0, SPSS, Inc., Chicago, IL, USA) was used for the analyses, and GraphPad Prism (Version 5.0, GraphPad Software, Inc., La Jolla, CA, USA) was used for figure generation. Continuous data are presented as the medians (range), and categorical data are presented as proportions. Parametric Student’s *t* tests were employed to evaluate continuous variables, while chi-square tests were used for categorical variables. All *P* values reported were two-sided, and a value of *P* < 0.05 was considered statistically significant.

## Results

**Fig. 1:**
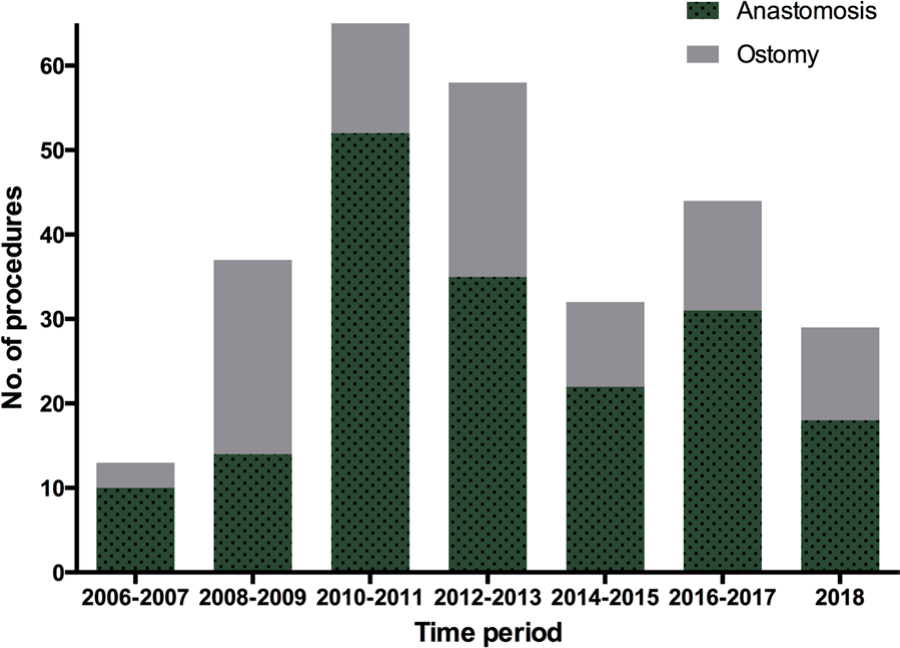
The number of bowel resections during debulking surgery in ovarian cancer patients at the Fudan University Shanghai Cancer Center over the past 13 years

In total, 282 ovarian cancer patients with advanced tumours received bowel resection as part of debulking surgery. Among them, 182 and 100 patients underwent anastomosis and ostomy formation, respectively. Figure 1 illustrates the number of bowel resections at our institution over the past 13 years. Table 1 presents patient information and surgery-related variables. For the entire cohort, the median age was 57 years (range, 23–92 years). The majority of patients had high-grade serous histology (88.7%). Neoadjuvant chemotherapy was administered in 49 (17.3%) patients. Ascites was present in 78.7% of the patients, and the median volume was 800 mL (range, 50–8000 mL). Extensive bowel resection and upper abdominal operation were performed in 29 (10.2%) and 69 (24.4%) patients, respectively. Three patients had protective stoma. The debulking results showed 121 (42.9%) patients with no gross residual disease and 248 (87.9%) with residual disease ≤ 1 cm. The median operation time was 197 min (range, 60–371 min), whereas the median blood loss was 1000 mL (range, 100–3500 mL). During the operation, 87.9% of patients received a transfusion, and the median volume transfused was three units (range, 1–11 units). For the whole population, the median time from surgery to discharge and chemotherapy was 13 days (range, 5–53 days) and 19 days (range, 7–50), respectively.

**Table 1:** Patient information and surgery-related outcomes

Variables	Total (n = 282)	Anastomosis (n = 182)	Ostomy (n = 100)	P
Age (years)	57 (23–83)	55 (25–77)	58.5 (26–83)	** < 0.01**
Body mass index (kg/m^2^)	22.2 (14.2–37.3)	22.0 (14.2–34.2)	23.2 (16.0–37.3)	0.10
Neo-adjuvant chemotherapy (%)	49 (17.4%)	29 (15.9%)	20 (20.0%)	0.39
Preoperative laboratory values				
CA-125 (U/mL)	1006.5 (3.5–31,803.7)	1114.5 (3.5–31,803.7)	907.3 (7.4–23,156.0)	0.55
Albumin (g/L)	40.0 (24.4–55.3)	40.7 (26.9–55.3)	39.6 (27.6–48.5)	0.64
Hemoglobin (g/L)	119 (62–151)	119 (76–151)	120 (62–147)	0.87
Postoperative day 1 laboratory values				
Albumin (g/L)	31.0 (19.3–48.9)	31.2 (19.9–48.9)	30.2 (19.3–43.5)	0.28
Hemoglobin (g/L)	111 (69–159)	111 (72–159)	111 (69–142)	0.16
High-grade serous carcinoma (%)	250 (88.7%)	157 (86.3%)	93 (93.0%)	0.09
Presence of ascites at surgery (%)	222 (78.7%)	147 (80.8%)	75 (75.0%)	0.26
Ascites volume (mL)	800 (50–8000)	800 (50–8000)	1000 (50–7500)	0.72
Extensive bowel resection (%)	29 (10.2%)	20 (11.0%)	9 (9.0%)	0.60
Upper abdominal surgery (%)	69 (24.4%)	57 (31.3%)	12 (12.0%)	** < 0.01**
Extent of debulking				
Residual disease = 0 cm (%)	121 (42.9%)	88 (48.4%)	33 (33.0%)	**0.01**
Residual disease ≤ 1 cm (%)	248 (87.9%)	166 (91.2%)	82 (82.0%)	**0.02**
Operation time (minutes)	197 (60–371)	203 (97–371)	172 (60–324)	** < 0.01**
Estimated blood loss (ml)	1000 (100–3500)	950 (100–3500)	1000 (200–2500)	0.98
Transfusion (%)	248 (87.9%)	156 (85.7%)	92 (92.0%)	0.12
Red blood cell transfusion (unit)	3 (0–11)	3 (0–11)	3 (0–9)	0.93
Postoperative hospital stay (days)	13 (5–53)	13 (5–53)	10 (5–40)	** < 0.01**
Time to chemotherapy (days)	19 (7–50)	20 (7–50)	18 (7–41)	0.14

*CA-125*  Cancer Antigen 125

We further compared the patient information and perioperative outcomes between the anastomosis and ostomy groups. Patients who received ostomy formation were significantly older than their anastomosis counterparts. The percentage of upper abdominal surgery was higher in the anastomosis group than in the ostomy group (31.3% vs. 12.0%, *P* < 0.001). More patients in the anastomosis group achieved complete (R0) or R1 resection. Not surprisingly, patients with anastomosis had both longer operation times (203 vs. 172 min, *P* = 0.002) and postoperative hospital stays (10 vs. 13 days, *P* < 0.001). However, no difference in the time interval from surgery to chemotherapy was noted between the two groups.

Overall, 29 patients received more than one-segment bowel resection, and the specific details are listed in Table 2. The rectosigmoid colon was the most commonly resected (268/320, 83.8%) followed by right hemicolectomy (19/320, 5.9%) and small bowel resection (9/320, 2.8%).

**Table 2:** Type of bowel resections

One-segment bowel resection (n = 253)	
Rectosigmoid resection	238
Right hemicolectomy	7
Ileocecal resection	2
Transverse colon resection	2
Left colon segmental resection	2
Left colon resection	2
Extensive bowel resection (n = 29)	
Rectosigmoid resection + small bowel resection	5
Rectosigmoid resection + ileocecal resection	3
Rectosigmoid resection + right hemicolectomy	10
Rectosigmoid resection + transverse colon resection	1
Rectosigmoid resection + left colon resection	4
Rectosigmoid resection + right colon segmental resection + small bowel resection	1
Rectosigmoid resection + right hemicolectomy + small bowel resection	1
Rectosigmoid resection + right hemicolectomy + left colon resection	1
Rectosigmoid resection + transverse colon segmental resection + left colon segmental resection	2
Rectosigmoid resection + left colon resection + small bowel resection	1
Rectosigmoid resection + right colon segmental resection + small bowel resection	1
Type of bowel surgery in descending order (n = 320)	
Rectosigmoid resection	268
Right hemicolectomy	19
Small bowel resection	9
Left colon resection	8
Ileocecal resection	5
Left colon segmental resection	4
Transverse colon resection	3
Right colon segmental resection	2
Transverse colon segmental resection	2

Table 3 shows the details on surgical complications. For the entire cohort, 23.0% (65/282) experienced complications to different extents. Of these complications, severe complications (CDC 3–5) accounted for 9.2% and mostly included pleural effusion requiring drainage (3.5%) followed by wound dehiscence requiring delayed repair in the operating room (1.8%). Notably, the surgical site infection (either superficial incisional, deep incisional, organ space or wound dehiscence) rate was 7.1% (20/282), including wound infection/dehiscence (3.2%, 9/282). Regarding anastomotic leakage, nine events were reported in total: one in the ostomy group (rectosigmoid resection + right hemicolectomy + left colon resection + ileostomy) and eight in the anastomosis group. The total number of bowel anastomoses in the entire population was 212, translating to an overall AL rate of 4.2% (9/212) per anastomosis. In the anastomosis group, the AL per patient was 4.4% (8/182), whereas the AL per anastomosis was 4.0% (8/202). Five patients with AL (four in the anastomosis group and one in the ostomy group) were successfully managed with conservative treatment. Overall, 187 patients had anastomosis after bowel resection: 162 with one-segment (162 anastomosis) and 25 with multiple bowel resections (50 anastomosis). The AL rate per patient was higher in patients with extensive bowel resection (8%, 2/25) than in those with one-segment resection (4.3%, 7/162). However, the AL per anastomosis rate was quite comparable between the two groups (4.3% vs. 4.0%). We further focused on patients with isolated rectosigmoid resection and anastomosis (n = 146). Among them, 119 had end-to-end anastomosis, whereas 27 had end-to-side anastomosis. Six patients (5.0%) in the end-to-end anastomosis group experienced AL, whereas no case was reported in the end-to-side group.

**Table 3:** Surgical complications

Mild complications in entire population (CDC 0–2)	39	13.8%
Bowel obstruction	15	5.3%
Infection (abdominal/pelvic/bloodstream)	8	2.8%
Wound infection/dehiscence	4	1.4%
Pleural effusion	4	1.4%
Heart arrhythmia	1	0.4%
Pancreatic leak	1	0.4%
Deep venous thrombosis	1	0.4%
Anastomotic leak with conservative treatment	5	1.8%
Severe complications in entire population (CDC 3–5)	26	9.2%
Pleural effusion requiring drainage	10	3.5%
Wound dehiscence requiring delayed repair in operation room	5	1.8%
Bowel obstruction	2	0.7%
Bleeding requiring return to operating room	2	0.7%
Septic shock	1	0.4%
Acute kidney failure	1	0.4%
Ureterostenosis requiring stent implantation in operation room	1	0.4%
Anastomotic leak requiring a second operation for intestinal ostomy	2	0.7%
Anastomotic leak leading to severe infection that requires intensive care unit stay	2	0.7%
Anastomotic leak		
Anastomotic leak in the entire population with anastomosis	9	4.2%^a^
Anastomotic leak in the anastomosis group	8	4.0%^a^
		4.4%^b^
Anastomotic leak in patients with one-segment bowel resection and anastomosis	7	4.3%^ab^
Anastomotic leak in patients with extensive bowel resection and anastomosis	2	4.0%^a^
		8.0%^b^
Anastomotic leak in patients with rectosigmoid resection only and anastomosis	6	5.0%^ab^

*CDC*  Clavien–Dindo Classification

^a^Anastomotic leak rate per anastomosis

^b^Anastomotic leak rate per patient

## Discussion

In the current series, we analysed the results of patients with advanced ovarian cancer receiving bowel resection in debulking surgery. In contrast to our previous two publications [9, 10], the current study included all patients who underwent bowel operations instead of isolated rectosigmoid resection. To the best of our knowledge, the present study is the first from a Chinese academic centre. All surgical procedures were performed by gynaecologic oncologists in our institution. We demonstrated that the complication rate in the study was comparable to that reported in the literature.

Ovarian cancer has different mechanisms of metastasis. According to a recent publication, parenchymal, peritoneal, and nodal metastasis accounted for 20.3%, 99.3%, and 39.3% of cases, respectively [25]. According to a previous study, 72% of advanced ovarian cancer patients had visible tumours in the small and large bowels [26]. The bowel resection rate during cytoreductive surgery ranged from 40 to 80% in institutions adopting radical surgery [11–13, 16]. A population-based study using SEER-Medicare examined 5,417 patients with advanced ovarian cancer diagnosed between January 2000 and December 2013 [27]. An increase in bowel resections was noted, and the total rate was 34% [27]. Two recent publications evaluated multiple bowel resections in ovarian cancer, including one from Germany [16] and one from Korea [22]. We noticed that the interval-debulking rate (IDS) was relatively lower in our study (17.3%) and other similar studies (no IDS in Peiretti et al. [28]; 19.3% in Berretta et al.[14]). The possible explanation for this might be that the bowel resection rate might be lower in patients after neoadjuvant chemotherapy.

A recent study included 4,965 debulking surgeries for ovarian cancer recorded in the American College of Surgeons' National Surgical Quality Improvement Program datasets (2006–2017) [29]. In the study, surgical site infection (superficial incisional, deep incisional, or organ space or wound dehiscence) was significantly more prevalent in the bowel resection/repair group (16.9% vs. 5.7%, p < 0.0001) [29]. They hypothesized that the high rate might be caused by inappropriate or insufficient antibiotic coverage in the setting of a high bacterial inoculum at the time of surgery [29].

Regarding perioperative adverse events, especially anastomotic leakage, we found that the overall AL rate was 4.2% per anastomosis. In our previous work including 50 cases receiving isolated rectosigmoid resection and anastomosis, the AL rate was 4.0% per patient. The AL rate was higher in patients with multiple bowel resections if the rate was calculated by patient (8.0%). However, there was no difference in the AL rate per anastomosis. Our reported AL rate is consistent with previous findings, as other series reported AL rates of 6.0% (Memorial Sloan-Kettering Cancer Center, USA) [11], 6.6% (eight hospitals in Spain) [19], 2.89% (Hopital Europeen Georges Pompidou, France) [30], and 6.9% (Comprehensive Cancer Center Vienna, Austria) [16].

Given the small number of patients with anastomotic leakage, we did not assess the underlying risk factors. A recent multicentre study from Spain, including 457 patients, investigated the risk factors for anastomotic leakage after colorectal resection in ovarian cancer patients [19]. They concluded that the following variables were independent risk factors for AL: age at surgery, preoperative serum albumin level, one or more additional small bowel resections, manual anastomosis and distance of the anastomosis from the anal verge [19]. Another study from the Mayo Clinic evaluated 42 AL cases compared to 84 controls with matched factors [31]. They found that multiple large bowel resections (rectosigmoid resection coupled with additional large bowel resection) were related to AL, and protective diverting stomas decreased the risk [31]. In our study, only three patients had protective stomas, whereas the AL rate was 4.2%. Therefore, we do not routinely perform protective stomas in our centre. We did pay attention to blood transfusion to ensure adequate blood supply and albumin supplementation as reflected by the pre- and postlaboratory parameters (Table 1). Out of curiosity, we compared the different types of anastomosis in patients with only rectosigmoid resection and anastomosis (n = 146). Interestingly, six patients (5.0%) in the end-to-end anastomosis group experienced AL, whereas no case was reported in the end-to-side group. However, due to the small sample size, we could not arrive at a conclusion.

The study has several limitations. First, it has inherent bias pertaining to its retrospective design. For example, clinical preoperative status (Eastern Cooperative Oncology Group Performance Status) was missing in some of the patients and thus not included in the analysis. Second, as mentioned before, we did not evaluate the risk factors for AL given the small number of outcome events. Third, we only assessed the perioperative outcomes, and survival information was not available. Finally, given that the study patients were recruited from a tertiary referral centre, the results might not be generalizable to all patients in China.

## Conclusions

Performance of bowel surgery in cytoreduction by experienced gynaecologic oncologists in a high-volume centre was feasible and resulted in a severe morbidity rate of 9.2%. Referrals should be considered at institutions where necessary treatments are unavailable.

## Data Availability

The datasets analyzed during the current study are not publicly available due to privacy or ethical restrictions. They are available from the corresponding author on reasonable request. Please contact Dr Libing Xiang.
